# The Relationship between Intelligence and Training Gains Is Moderated by Training Strategy

**DOI:** 10.1371/journal.pone.0123259

**Published:** 2015-04-10

**Authors:** Hyunkyu Lee, Walter R. Boot, Pauline L. Baniqued, Michelle W. Voss, Ruchika Shaurya Prakash, Chandramallika Basak, Arthur F. Kramer

**Affiliations:** 1 Brain Plasticity Institute, Posit Science, San Francisco, California, United States of America; 2 Department of Psychology, Florida State University, Tallahassee, Florida, United States of America; 3 Beckman Institute for Advanced Science and Technology, University of Illinois at Urbana-Champaign, Urbana, Illinois, United States of America; 4 Department of Psychology, The Univeristy of Iowa, Iowa City, Iowa, United States of America; 5 Department of Psychology, The Ohio State University, Columbus, Ohio, United States of America; 6 Center for Vital Longevity, University of Texas at Dallas, Richardson, Texas, United States of America; The University of Chicago, UNITED STATES

## Abstract

We examined the relationship between training regimen and fluid intelligence in the learning of a complex video game. Fifty non-game-playing young adults were trained on a game called Space Fortress for 30 hours with one of two training regimens: 1) Hybrid Variable-Priority Training (HVT), with part-task training and a focus on improving specific skills and managing task priorities, and 2) Full Emphasis Training (FET) in which participants practiced the whole game to obtain the highest overall score. Fluid intelligence was measured with the Raven’s Progressive Matrix task before training. With FET, fluid intelligence was positively associated with learning, suggesting that intellectual ability played a substantial role in determining individual differences in training success. In contrast, with HVT, fluid intelligence was not associated with learning, suggesting that individual differences in fluid intelligence do not factor into training success in a regimen that emphasizes component tasks and flexible task coordination. By analyzing training effects in terms of individual differences and training regimens, the current study offers a training approach that minimizes the potentially limiting effect of individual differences.

## Introduction

With the increasing complexity of skills required in modern society, it has become an important issue to provide efficient training regimens to expedite learning and increase the level of mastery of complex tasks. In examining the effectiveness of training regimens, individual differences in cognitive abilities should be considered [1–7]. Individuals may respond to different forms of training in different ways as their abilities to process and apply information vary. In fact, several video game training studies have observed that the effect of training was modulated by pre-existing individual differences such that individuals with the lowest initial scores gained the most from the training [2, 6, 8]. Moreover, individual differences in initial task performance interact with training regimens. Video game training with younger adults has shown that participants trained to flexibly manage task priorities (Hybrid Variable Priority Training, HVT) showed better game performance than participants trained to treat all task components equivalently (Full Emphasis Training, FET). Interestingly, the benefit from HVT was largest for those participants who started training with lower game proficiency, and the specific training regimen mattered less for participants who started with high game proficiency [6].

The association between individual differences and the effect of training regimens is not always consistent. With mnemonic memory strategy instruction, some studies find a positive association between individual cognitive abilities and training gains [9–11], while others find negative associations [12, 13]. These mixed findings may be explained by the magnification and compensation accounts of training [7]. According to this view, initial strategy instruction compensates for inefficient processing mostly among the less proficient individuals, while individuals with high cognitive abilities show little benefit from initial strategy instruction since they are already functioning at a high level, resulting in a negative association between individual differences and performance gains (compensation account). Meanwhile, with extended practice without specific instruction, individual differences in cognitive abilities are revealed, resulting in a positive association between individual differences and performance gains (magnification account). These accounts suggest that specific training strategy may either facilitate or inhibit learning depending upon the characteristics of different learners and the length of the training.

In the current study, we examined the effect of individual differences in fluid intelligence, training regimens, and their interaction on the success of 30 hours of video game training (15 sessions, 2 hours for each session). Participants played the Space Fortress video game [14] for 30 hours using one of two training regimens: Hybrid Variable Priority Training (HVT), which included part task training and task management and coordination training, or Full Emphasis training (FET) in which participants simply practiced the game to obtain the highest overall score. Game training results were initially presented in Lee et al. (2012) [6], which found that HVT led to better mastery of Space Fortress, with high levels of retention over seven months. However, in the previous analysis, Lee and colleagues examined the overall group training effect and did not consider the role of individual differences in fluid intelligence, and the interaction between training regimens and individual differences.

We hypothesized, based on the magnification and compensation model, that with HVT, participants with lower fluid intelligence would compensate for individual differences with strategy training, therefore resulting in a null or negative association between performance improvements and fluid intelligence. In contrast, with FET, participants with high fluid intelligence would maintain superior performance throughout training, thus leading to a positive association between individual differences in fluid intelligence and performance improvements.

## Material and Methods

### Participants

Fifty participants (ages 18–30, 19 males) were recruited from the Urbana-Champaign community and were paid fifteen dollars per hour for completing testing and training. Twenty-five participants were assigned to the Hybrid Variable-Priority Training group (HVT), and twenty-five were assigned to the Full Emphasis Training group (FET). Initially participants were randomly assigned to each group. Halfway through recruitment, demographic characteristics of each group were checked and used as a guideline for group assignment to ensure that groups did not differ in terms of gender or age. Demographics are presented in Table 1. The University of Illinois Internal Review Board (IRB) approved this study, and all participants provided written informed consent according to the principles of the Declaration of Helsinki.

**Table 1:** Descriptive characteristics of participants in FET and HVT. Standard deviations are within parentheses.

	Full Emphasis Training (FET)	Hybrid Variable-Priority Training (HVT)
**N (number of male)**	25 (9)	25 (10)
**Age**	21.91 (2.77)	20.88 (2.06)
**Years of Education**	15.52 (2.19)	14.68 (1.85)
**Baseline Score**	-844.45(2086.81)	-1034.78 (1907.14)

### Training Video Game: Space Fortress

**Fig 1:**
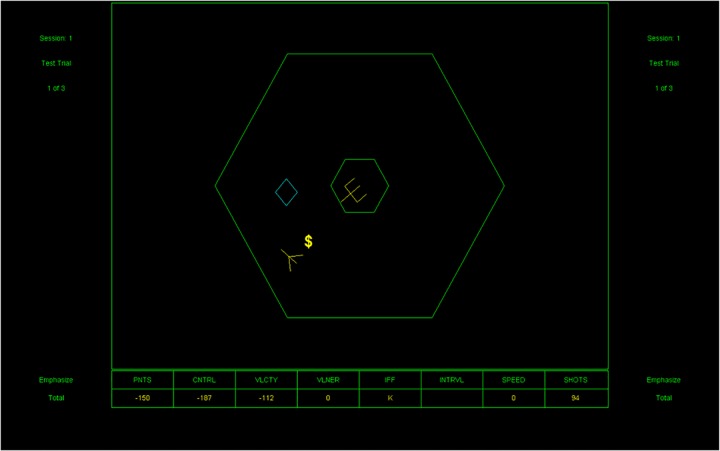
Screenshot of Space Fortress game.

Space Fortress (see Fig 1) requires players to navigate their ship with precise control using a joystick. The main goal of the game is for players to destroy the Space Fortress (located at the center of the screen) while avoiding damage to their own ship and dealing with mines and bonus symbols. A complete description of Space Fortress game is presented in Donchin (1989) [14].

### Training Regimens

Each session started and ended with 3 game trials (which will be referred to as a pre and post test block) in which participants in both of the training groups were asked to maximize performance and focus on obtaining the highest total score by emphasizing each task component equally. In between the test blocks, there was a practice block. For the HVT group, the practice block of the first 5 sessions was a combination of part-task training and Variable Priority Training (VPT). Part-task training was used to train the components of the game independently, starting from the simple sub-task to the full game [15]. VPT focused on emphasizing different components of the overall task within the context of the whole Space Fortress task. Participants were asked to focus on improving and monitoring different sub-scores of the game during practice [14]. After the first 5 sessions, participants completed 10 sessions of VPT during the practice block. For the FET group, throughout the 15 sessions, participants were always asked to maximize total score during practice. A complete description of each training regimen is presented in Lee et al (2012) [6].

### Baseline Fluid Intelligence measurement

**Fig 2:**
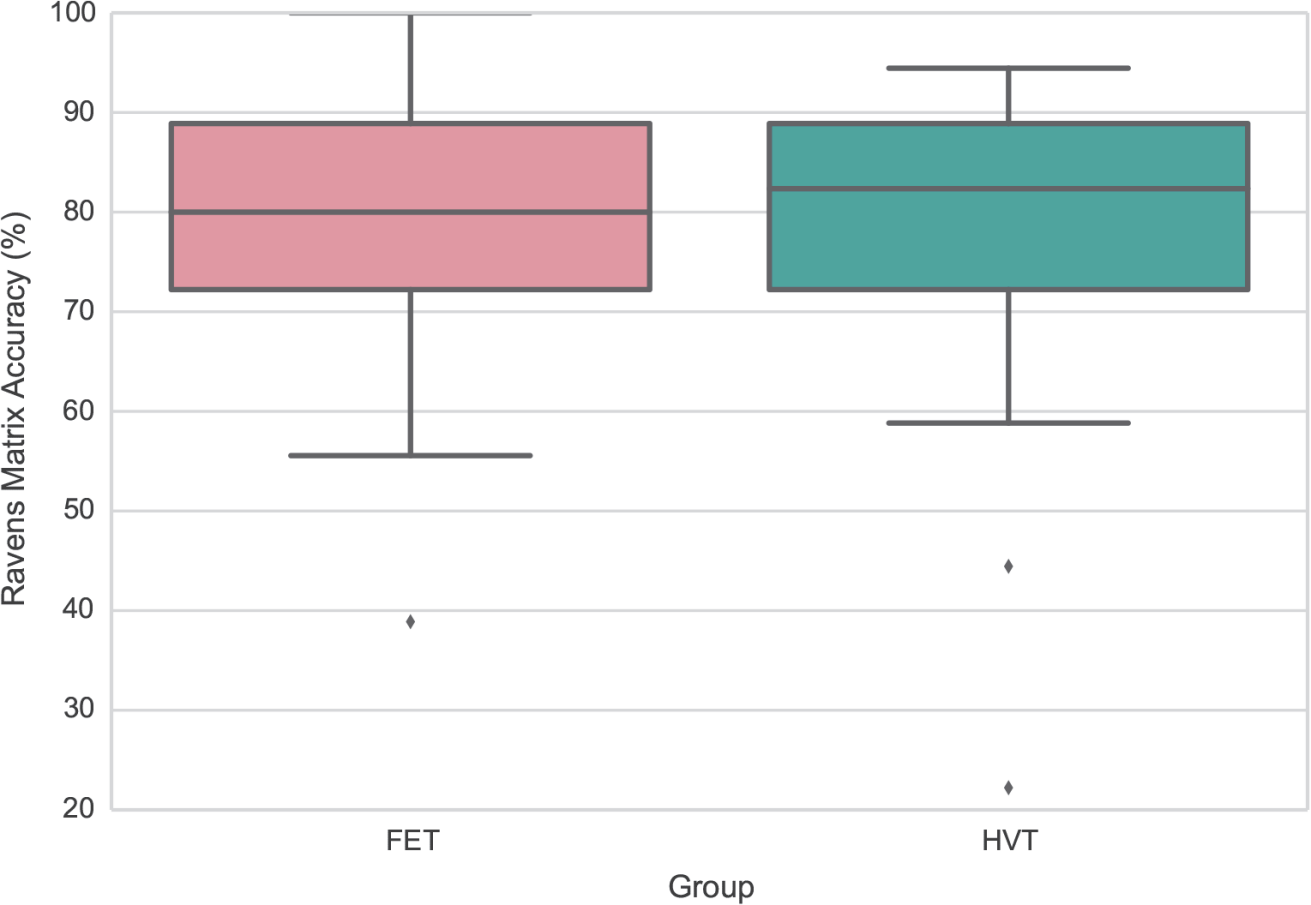
Box-plots for Raven’s progress matrix scores. There was no difference between FET and HVT in Raven’s progressive matrix scores.

Participants completed the Raven’s Progressive Matrices task [16] before initiating SF training. In the Raven’s Progressive Matrices task, participants were presented with a complex visual pattern with a piece cut out of it and asked to find the missing piece that completed the pattern. Participants were given 5 min to complete a practice version of the test, and then given 30 min to complete 18 test items. Accuracy was used as a primary measure, and there was no significant difference in accuracy between FET and HVT groups, F(1, 48) = .07, *p* >.05. The box plot of accuracy distribution of each group is presented in Fig 2.

### Training Gains

**Fig 3:**
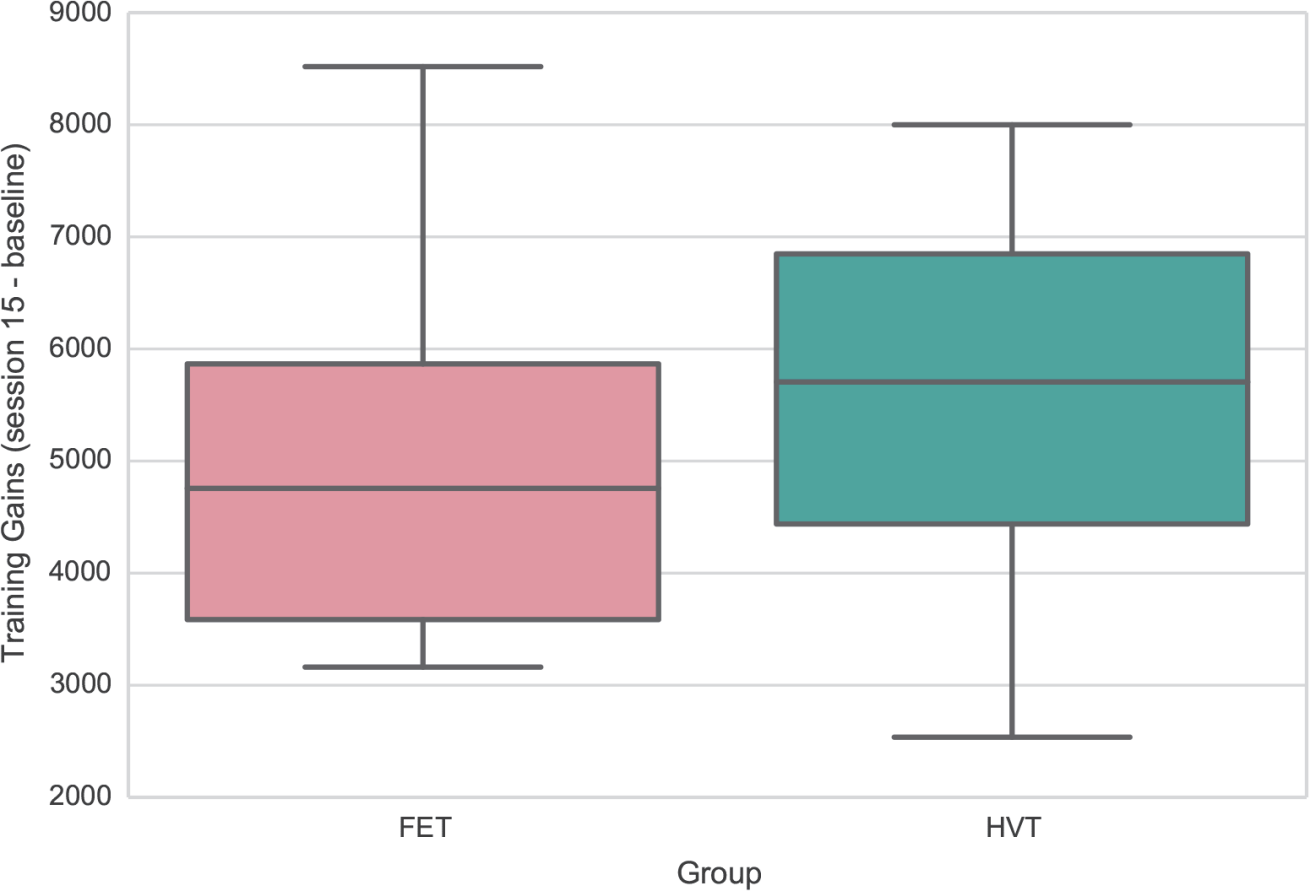
Box plots for training gains for each group. HVT showed higher training gains compared to FET.

Training gains were measured by the score difference between final performance (post test block of session 15) and baseline (pre test block of session 1). We performed an ANOVA with training gain as dependent variable, and training regimen as between-subjects variable. Gender was included as a covariate to address gender effects in video game play [17]. Baseline performance was also included as a covariate to account for ceiling effects in performance improvement. Results showed higher training gains in the HVT group compared to the FET group, F(1,46) = 4.73, *p* <.05. The box plot of training gain distribution of each group is presented in Fig 3.

## Results

To examine whether training regimen moderates the relationship between fluid intelligence and training gain, we conducted a hierarchical multiple regression analysis. Training regimen, fluid intelligence, baseline performance and gender were included as predictors, and these variables accounted for a significant amount of variance in Space Fortress training gain, R^2^ = .533, *F*(4, 45) = 12.841, *p* < .01. The variables were then centered to address potential multi-collinearity with the interaction term, which was created using training regimen and fluid intelligence [18]. The interaction term was added to the regression model and accounted for a significant amount of variance in training gain, ΔR^2^ = .046, ΔF (1, 44) = 4.76, *b* = -43.98, *t*(44) = -2.184, *p* = .034.

**Fig 4:**
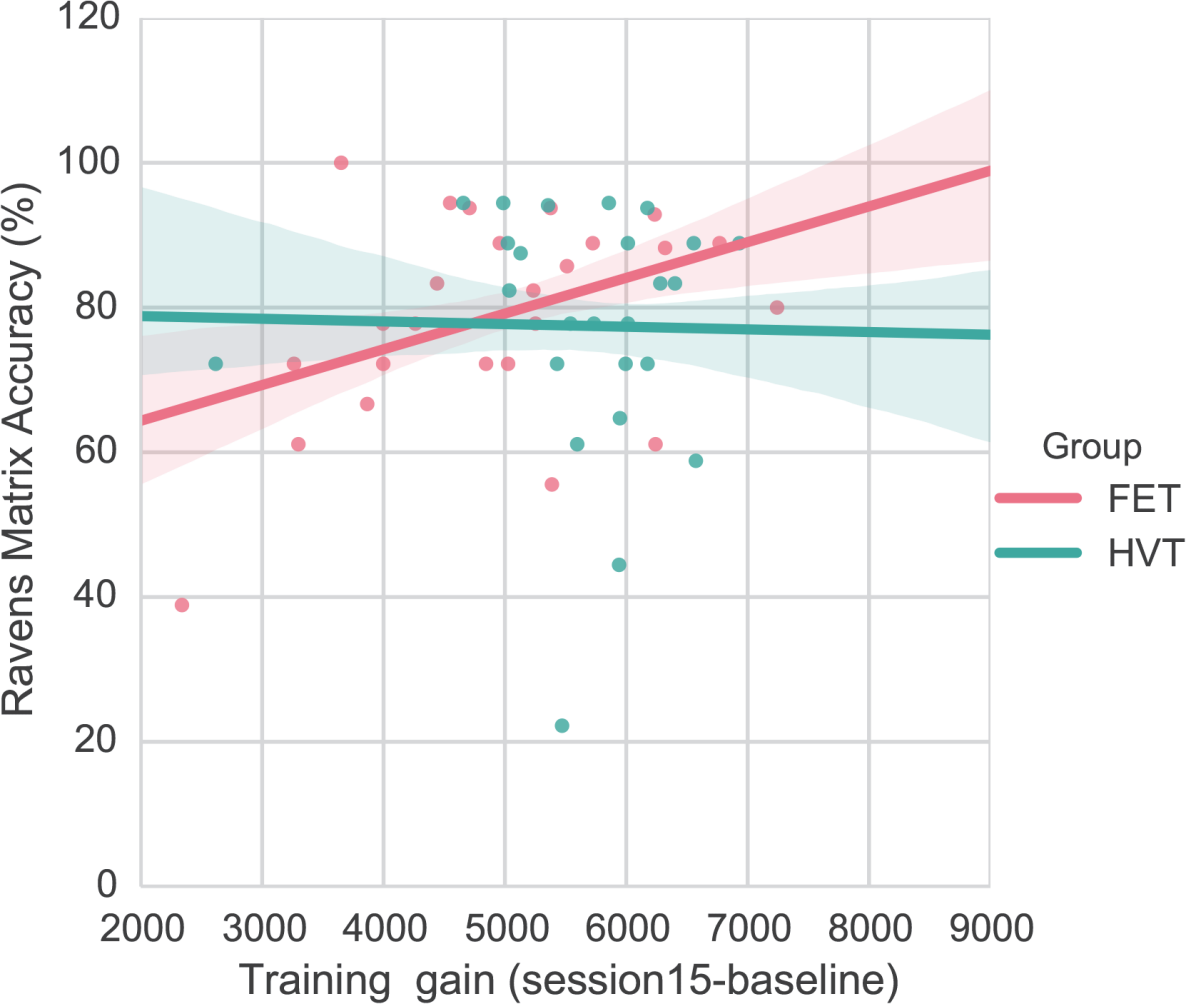
Partial correlation between baseline fluid intelligence and training gains, while controlling for gender and baseline performance. Bands represent the standard error of an estimate.

Post-hoc examination of the correlation between training gain and fluid intelligence showed that the direction of the effect differed for the two training groups (*r* = .408, *p*<.05 for FET, *r* = -.023, *p* = n.s. for HVT, see Fig 4). In FET, participants with higher baseline fluid intelligence improved more than participants with lower fluid intelligence. In contrast, in HVT, there was no difference on performance improvement between participants who started with lower and higher fluid intelligence.

**Fig 5:**
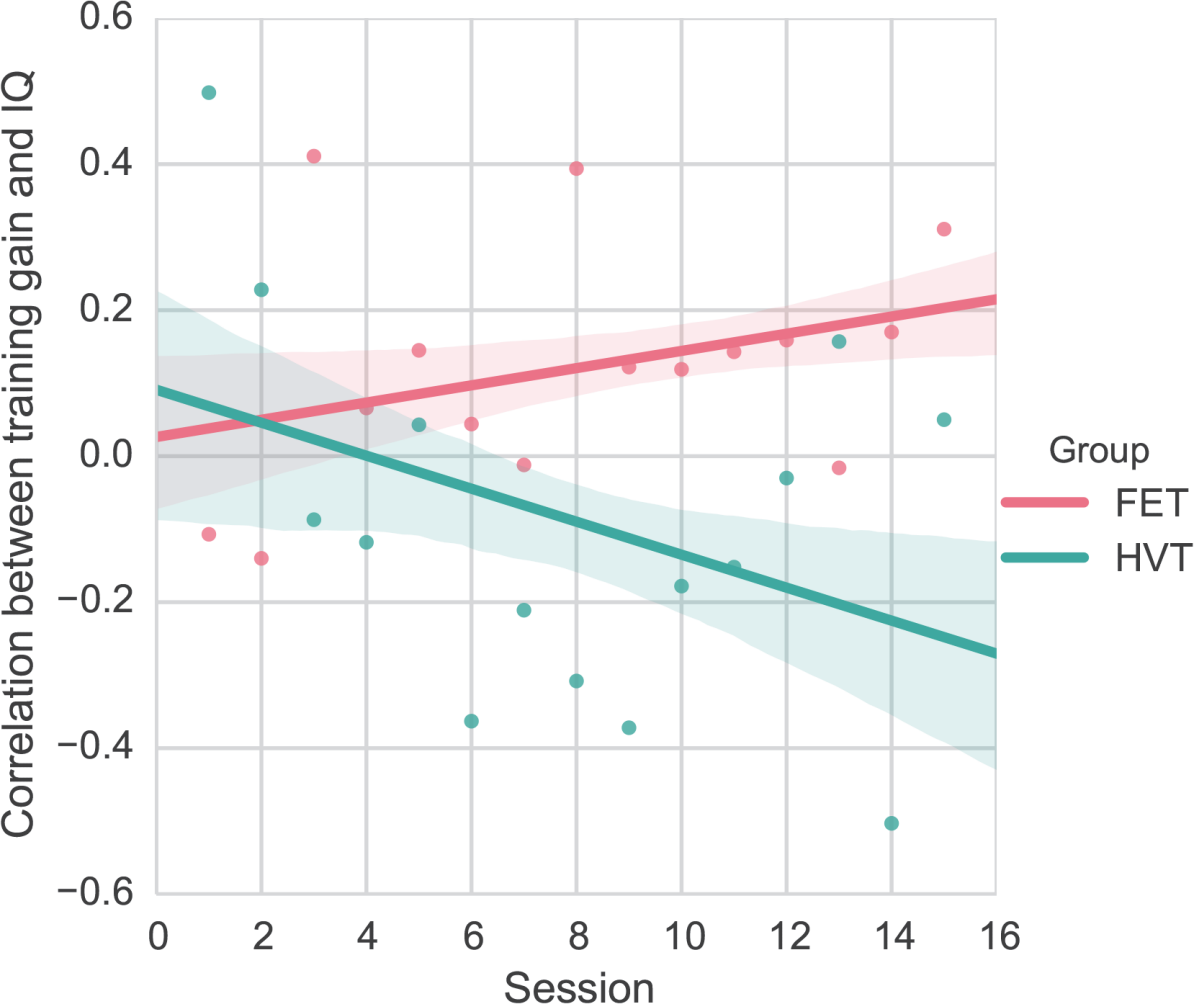
Partial correlation between baseline fluid intelligence and training gains in each session, while controlling for gender and baseline performance. Bands represent the standard error of an estimate.

When we examined the relationship between fluid intelligence and game improvement across sessions (measured by the score difference between the pre and post test block of each session), we found that extended practice with FET magnified individual differences in fluid intelligence. In contrast, extended practice with HVT reduced individual differences in fluid intelligence. Hierarchical multiple regression analysis revealed that training regimen and session accounted for a significant amount of variance in the correlation between fluid intelligence and training gain, R^2^ = .212, *F*(2,27) = 3.62, *p* < .05. The centered interaction term using training regimen and session was added to the regression model and accounted for a marginally significant amount of variance in the correlation between fluid intelligence and training gain, ΔR^2^ = .101, ΔF (1, 26) = 3.80, *b* = -.034, *t*(26) = -1.950, *p* = .062 (see Fig 5).

## Discussion

In this study, we observed that the association between individual differences in fluid intelligence and training gains in a complex video game (Space Fortress) is modulated by training regimen. In the FET group, fluid intelligence positively predicted learning in the Space Fortress game. In the HVT group, there was no association between fluid intelligence and learning, suggesting that HVT training might reduce the role of pre-existing individual differences in fluid intelligence determining task performance.

These results are consistent with the compensation and magnification model [7]. With guidance provided by training strategy (HVT), individuals with lower fluid intelligence gain as much as those who with higher fluid intelligence (compensation). In contrast, without specific guidance on game play (FET), extended training enhances performance difference between low and high intelligence participants (magnification).

The main difference between HVT and FET is that HVT is more structured with part-task training (teaching the game with manageable chunks) and variable priority training (providing chances to explore different aspects of game). In terms of learning, FET can be more challenging because it requires participants to determine the best learning method on their own. In this learning condition, individuals with high fluid intelligence may exhibit better performance because they may be more likely to devise and implement alternative strategies for completing tasks, and as such, learn to play the game better overall. In contrast, in the learning condition where specific training approaches are provided, individual differences in fluid intelligence might play a smaller role since all participants are given the same approach to playing the game.

One possible consideration with the different instructions given to HVT and FET is experimenter demand, given the different instructions issued to the two groups. In terms of instruction time, HVT participants received more instruction only during the part-task training blocks (the first hour and 10 minutes of initial 5 sessions of training). HVT participants were not aware that they were received more instruction than the FET group in the first five sessions, however. Although there was a difference in instruction between the training groups in the practice blocks, participants in both groups received the same instruction during “test blocks” (*and only scores from test blocks are used for analysis*) asking them to maximize performance and focus on obtaining the highest total score by emphasizing each task component equally. Instructions given to HVT and FET are presented in S1 Table.

The interaction between training regimen and individual differences is also shown in a recent study that tested the influence of fitness on memory in children [19]. Higher fit 9–10 year old children displayed higher memory performance than lower-fit children in verbal-spatial learning memory tasks. Interestingly, the fitness-based memory performance difference between the higher and lower fit children was reduced when guided study instruction was provided. Taken together, these findings suggest that effective training strategies can be used to overcome individual differences that are normally associated with poorer performance.

The superiority of HVT in minimizing pre-existing individual differences provides insight into the effect of practice on training success in general. A recent meta-analysis revealed that deliberate practice explained only 26% of the variance in performance for games [20], suggesting that basic abilities and other individual difference factors may explain additional variance in learning and performance. We have shown from the current results that individual differences in fluid intelligence play an important role in determining the learning from practice with FET. However, with a more structured training regimen such as HVT, practice could have individuals with lower fluid intelligence perform as proficiently as those with higher fluid intelligence on the trained task [21], suggesting that not only individual differences, but also training regimen should be included in the discussion of the relation between practice and training success.

Addressing individual differences in learning from interventions and their interaction with training regimens is particularly important for identifying or developing a more effective training program tailored to an individual’s characteristics and needs. Since only healthy young adults were investigated in this study, our results may not generalize to other populations. However, the interaction between fluid intelligence and training regimens suggests that directed training strategies such as a combination of part-task training and variable priority training may be more useful to overcome the individual differences in fluid intelligence.

In sum, the findings described herein provide insight into designing cognitive training protocols that maximize training gain and minimize the influence of individual differences. Future research should focus on identifying sensitive metrics of individual differences, such as how different neural networks are associated with individual differences, training regimens, and their interaction.

## Supporting Information

S1 Table Instructions used in the study. (DOCX)
