# Supplementary material for: The Relationship between Intelligence and Training Gains Is Moderated by Training Strategy
Source: PLoS One. 2015 Apr 10;10(4):e0123259. doi: 10.1371/journal.pone.0123259 (PMC4393125; doi:10.1371/journal.pone.0123259)
Supplement: S1 Table — (DOCX) [file pone.0123259.s001.docx]

| Group | Instruction |
| --- | --- |
| Test Block (common to FET and HVT) | All test trials will highlight the TOTAL SCORE. To maximize your TOTAL SCORE, you should do as well as you can on all four sub-scores: the Velocity sub-score, the Control sub-score, the Speed sub-score and the Points sub-score. |
| FET (Fixed Emphasis Training) | This practice session will highlight the TOTAL SCORE. To maximize your TOTAL SCORE, you should do as well as you can on all four sub-scores: the Points sub-score, the Control sub-score, the Velocity sub-score, and the Speed sub-score. |
| HVT (Part-training) | The object of this task is to destroy the fortress as many times as possible within the given amount of time. You will not be able to rotate the ship; it will automatically be facing the fortress and all you need to do is fire at it. The fortress will not be able to turn or fire. |
|  | The object of this task is to stop the spaceship as quickly as possible. The spaceship will be moving in a random direction at a random velocity. Each time the ship is stopped, it will shoot off in a new direction at a new velocity, and you are to stop the spaceship again. |
|  | The object of this task is to aim the spaceship at the fortress so that the missiles hit it. The spaceship will be automatically firing and moving, the only thing you will be able to do is rotate the ship so it is aimed at the fortress. The fortress will not be firing at you. |
|  | The object of this task is to shoot the fortress as many times as possible without hitting it with a double shot. The ship will be moving across the screen at various distances and all you will be able to do is rotate the ship to aim it at the fortress and fire at it. The fortress will not shoot at you. |
|  | The object of this task is to navigate around the corner of the tracks without hitting any walls so that the spaceship crosses over the target. You can only rotate the ship before it reaches the thrust area, therefore you must carefully plan how you want to turn the ship before it reaches the thrust area. Once you reach the thrust area, you may thrust forward as many times as you need to make the turn before you exit the thrust area. |
|  | The object of the next task is to successfully turn through the angle on the track so that the spaceship passes over the target at the end of the track. There will be three different tracks to navigate. The ship will be facing almost backwards at the beginning of each trial and you will not be able to rotate it. The only thing you need to do is apply thrust to the ship once it enters the thrust area so that the ship passes over the target under the critical velocity. Remember, you can apply thrust more than once if you need to, as long as all the thrusts occur in the thrust area. |
|  | The object of the next task is to navigate around a corner of the hexagon track without hitting any walls while trying to hit the fortress with as many missiles as possible. The ship will automatically be firing, so you just have to aim at the fortress. The fortress will not shoot at you. A target will indicate which corner of the track you are to navigate around for each trial. You will only be given a short amount of time to navigate the corners of the hexagon, so do not hesitate too long. |
|  | This task is identical to the one you just completed except now the hexagon size is smaller than the previous one. You have to navigate around a corner of the hexagon track without hitting any walls while trying to hit the fortress with as many missiles as possible. The ship will automatically be firing, so you just have to aim at the fortress. The fortress will not shoot at you. A target will indicate which corner of the track you are to navigate around for each trial. You will only be given a short amount of time to navigate the corners of the hexagon, so do not hesitate too long. |
|  | The object of this task is to navigate the spaceship around the hexagon track without hitting any walls while aiming at the fortress. The spaceship will be firing automatically. You must pass over the corners of the hexagon track in numerical order to score points. If you hit a wall, your ship will return to starting position and you must start your point accumulation over. The fortress will not be firing at you. |
|  | This task is just like the one you just completed except now the spaceship will not fire automatically. Now you must fire at the fortress yourself and try to destroy it as many times as possible. |
|  | This task is identical to the one you just completed except now the fortress will track your ship and fire at it. You must navigate the hexagon track in numerical order, destroy the fortress as many times as possible, and avoid being shot by the fortress. |
|  | When a mine appears, check the letter under IFF. If the mine is a friend, aim and shot it. If it is a foe, press the right botton on the mouse twice with the interval between each button press between 250-400 msec, then aim and shot it. Let the mine comes to the ship, then turn and fire when they are close. |
|  | Underneath the Fortress, different symbols will appear. When a '$' appears twice in a row, you have the opportunity to obtain more resources. You can choose to get up to 50 missiles, or 100 more points. The choice is yours.  Select more points by pressing the left button on the mouse.  Select more missiles by pressing the middle button on the mouse. |
|  | You destroy the fortress as many times as possible within the given amount of time.  When a mine appears, check the letter under IFF. If the mine is a friend, aim and shoot it. If it is a foe, press the right button on the mouse twice with the interval between each button press between 250-400 msec, then aim and shot it. |
| HVT (Variable Priority Training) | This practice session will highlight the TOTAL SCORE. To maximize your TOTAL SCORE, you should do as well as you can on all four sub-scores: the Points sub-score, the Control sub-score, the Velocity sub-score, and the Speed sub-score. |
|  | This practice session will highlight the VELOCITY sub-score. To maximize your VELOCITY sub-score, move the ship at a low velocity. You should play the whole game, but you should focus your attention on the VELOCITY sub-score so that you do as well as you can on it. You should do as well as possible on the other sub-scores without allowing the VELOCITY sub-score to suffer. |
|  | This practice session will highlight the CONTROL sub-score. To maximize your CONTROL sub-score you should stay on the screen and move clockwise within the hexagonal boundaries. You should play the whole game, but you should focus your attention on the CONTROL sub-score so that you do as well as you can on it. You should do as well as possible on the other sub-scores without allowing the CONTROL sub-score to suffer. |
|  | This practice session will highlight the POINTS sub-score. To maximize your POINTS sub-score, you should destroy the Fortress as many times as possible; correctly identify and destroy as many mines as possible; avoid letting your ship get hit or destroyed by the Fortress or mines; select the bonus points whenever possible except when the shots counter is below 50. You should play the whole game, but you should focus your attention on the POINTS sub-score. |
|  | This practice session will highlight the SPEED sub-score. To maximize your SPEED sub-score, you should destroy mines quickly and appropriately. You should play the whole game, but you should focus your attention on the SPEED sub-score so that you do as well as you can on it. You should do as well as possible on the other sub-scores without allowing the SPEED sub-score to suffer. |
